# The role of advanced endoscopy in appendiceal polyp management and outcomes

**DOI:** 10.1007/s00464-024-10726-w

**Published:** 2024-03-04

**Authors:** Carla F. Justiniano, Ilker Ozgur, David Liska, Michael A. Valente, Scott R. Steele, Emre Gorgun

**Affiliations:** Department of Colorectal Surgery, Digestive Disease and Surgery Institute, Cleveland Clinic, 9500 Euclid Avenue, Cleveland, OH 44195 USA

**Keywords:** Advanced endoscopy, Appendiceal orifice polyps, EMR, ESD

## Abstract

**Background:**

Appendiceal orifice lesions are often managed operatively with limited or oncologic resections. The aim is to report the management of appendiceal orifice mucosal neoplasms using advanced endoscopic interventions.

**Methods:**

Patients with appendiceal orifice mucosal neoplasms who underwent advanced endoscopic resections between 2011 and 2021 with either endoscopic mucosal resection (EMR), endoscopic mucosal dissection (ESD), hybrid ESD, or combined endoscopic laparoscopic surgery (CELS) were included from a prospectively collected dataset. Patient and lesion details and procedure outcomes are reported.

**Results:**

Out of 1005 lesions resected with advanced endoscopic techniques, 41 patients (4%) underwent appendiceal orifice mucosal neoplasm resection, including 39% by hybrid ESD, 34% by ESD, 15% by EMR, and 12% by CELS. The median age was 65, and 54% were male. The median lesion size was 20 mm. The dissection was completed piecemeal in 49% of patients. Post-procedure, one patient had a complication within 30 days and was admitted with post-polypectomy abdominal pain treated with observation for 2 days with no intervention. Pathology revealed 49% sessile-serrated lesions, 24% tubular adenomas, and 15% tubulovillous adenomas. Patients were followed up for a median of 8 (0–48) months. One patient with a sessile-serrated lesion experienced a recurrence after EMR which was re-resected with EMR.

**Conclusion:**

Advanced endoscopic interventions for appendiceal orifice mucosal neoplasms can be performed with a low rate of complications and early recurrence. While conventionally lesions at the appendiceal orifice are often treated with surgical resection, advanced endoscopic interventions are an alternative approach with promising results which allow for cecal preservation.

Approximately 25% of colorectal resections for neoplastic disease in the United States (US) are for non-malignant colorectal polyps, and the incidence of colectomies and proctectomies performed for these benign polyps is on the rise [1]. Although laparoscopic colorectal resection have improved outcomes as compared to open, they still are associated with significant morbidity and mortality in addition to the potential for long-term functional implications [2]. As such, the adaptation of advanced endoscopic techniques for resection of polyps not amenable to conventional endoscopic removal merits further study in the US to potentially avoid such a high rate of oncologically unnecessary colorectal resections with their associated complications and costs [3, 4]. While the advanced endoscopic techniques of endoscopic mucosal resection (EMR) and endoscopic submucosal dissection (ESD) have evolved over the last two decades with a growing body of literature demonstrating efficacy and safety internationally [5, 6], the data are sparse among US patients with colon and rectal lesions [7]. Furthermore, the use of such techniques in the cecum, and particularly for appendiceal orifice (AO) lesions, poses unique technical challenges. For instance, these lesions can have submucosal fibrosis secondary to previous inflammation [8, 9]. Moreover, the AO tends to be visualized face-on in front of the endoscope with the tip of the operating tool often perpendicular to the dissection field which can increase the risk of perforation. Additionally, paradoxical endoscope movement in the cecum and peri-appendiceal area can occur making this location one that can be difficult to address [8, 9]. To date, only limited case studies or small series have explored advanced endoscopic technique use for AO lesions. The goal of the present study is to describe the management of appendiceal orifice mucosal neoplasms (AOMN) with advanced endoscopic techniques in a cohort of prospectively captured patients. The hypothesis was that AOMN could be safely resected with low complication rates allowing for organ preservation.

## Materials and methods

This study was approved by our institutional review board. We analyzed prospectively collected data of consecutive patients with mucosal lesions involving the AO who underwent advanced endoscopic techniques between 2011 and 2021 at our quaternary care hospital. All patients had been referred for management of presumed benign neoplasms not amenable to conventional endoscopic removal. Patients underwent either endoscopic mucosal resection (EMR), endoscopic mucosal dissection (ESD), hybrid ESD (combines elements of both EMR and ESD), or combined endoscopic laparoscopic surgery (CELS). Based on pre-procedure information, cases which were deemed a priori by the surgeon to be more difficult due to location, size, and/or characteristics were scheduled in the operating room with the option for CELS rather than pure ESD/EMR. These patients are consented for CELS at time of their initial informed consent.

Patient factors of interest included age, sex, BMI, and ASA. Lesion factors of interest included size (greatest diameter), Paris type, and Kudo classification. Procedure details included were length of procedure, location of the procedure (endoscopy suite versus operating room), technique utilized, and whether the specimen was resected en bloc or piecemeal. Overall procedure time is defined as the time from the beginning to the end of the procedure. Dissection time was reported as the time from the beginning to the end of the dissection. Outcomes of interest were final pathology, discharge, 30-day complications, and recurrence. The standard surveillance recommendation is a repeat colonoscopy at 6 months; given our referral patterns, many patients return to their local gastroenterologist for this repeat colonoscopy. Most data values are described as frequency (percentage). Continuous variables are presented as medians with interquartile range (IQR).

### Technical considerations

**Fig. 1:**
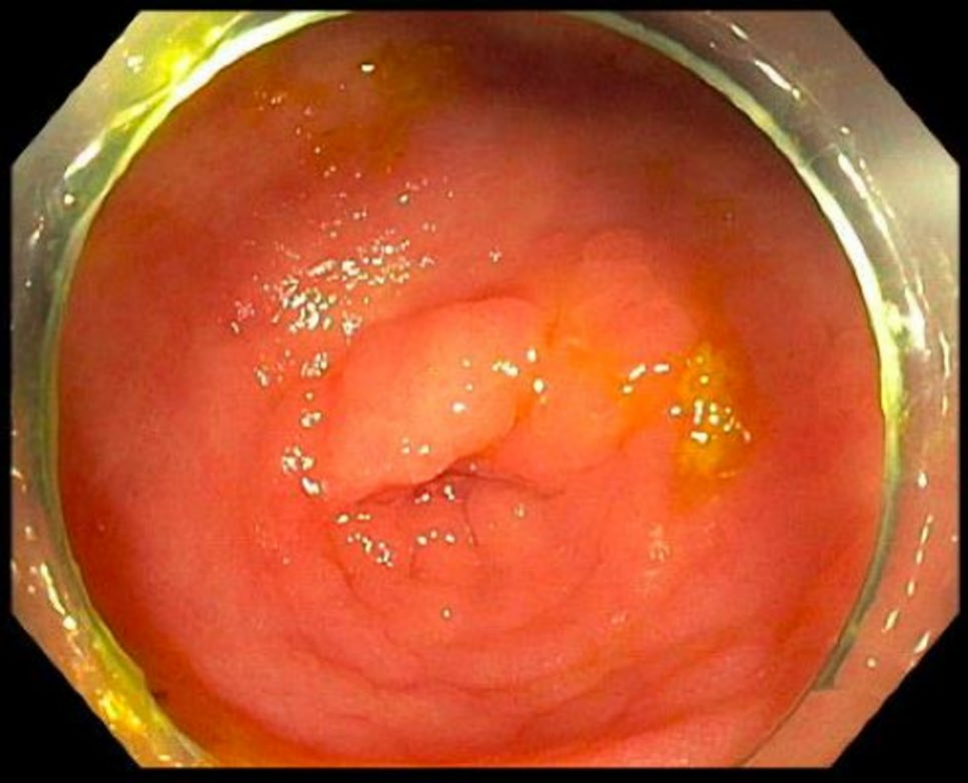
Appendiceal orifice lesion under normal light

**Fig. 2:**
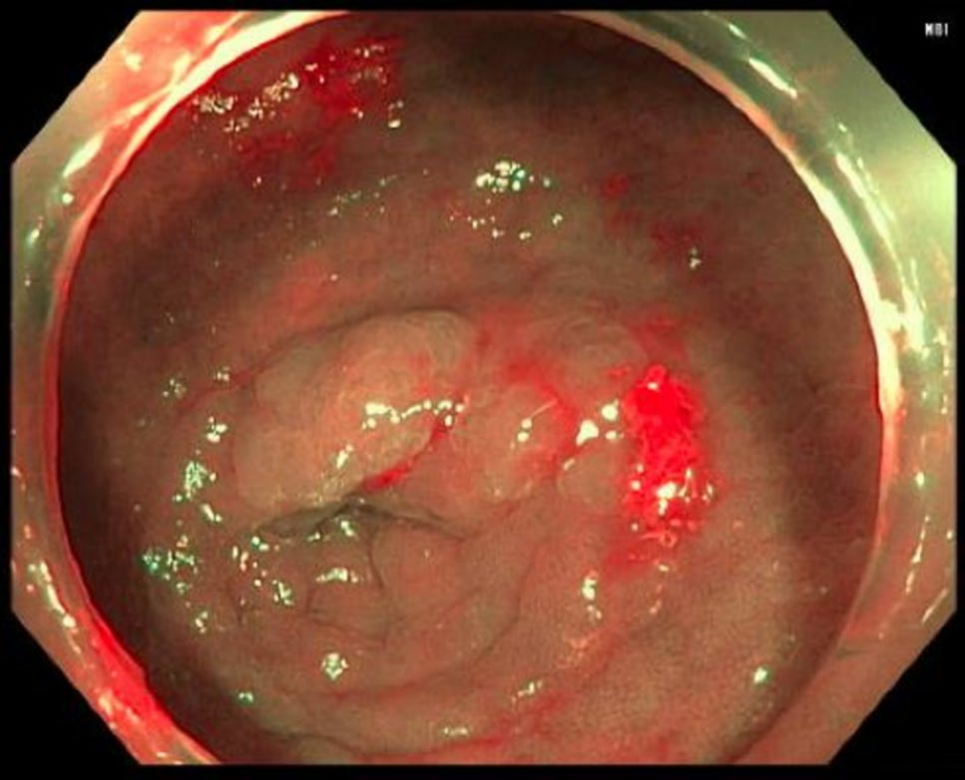
Appendiceal orifice lesion under NBI

**Fig. 3:**
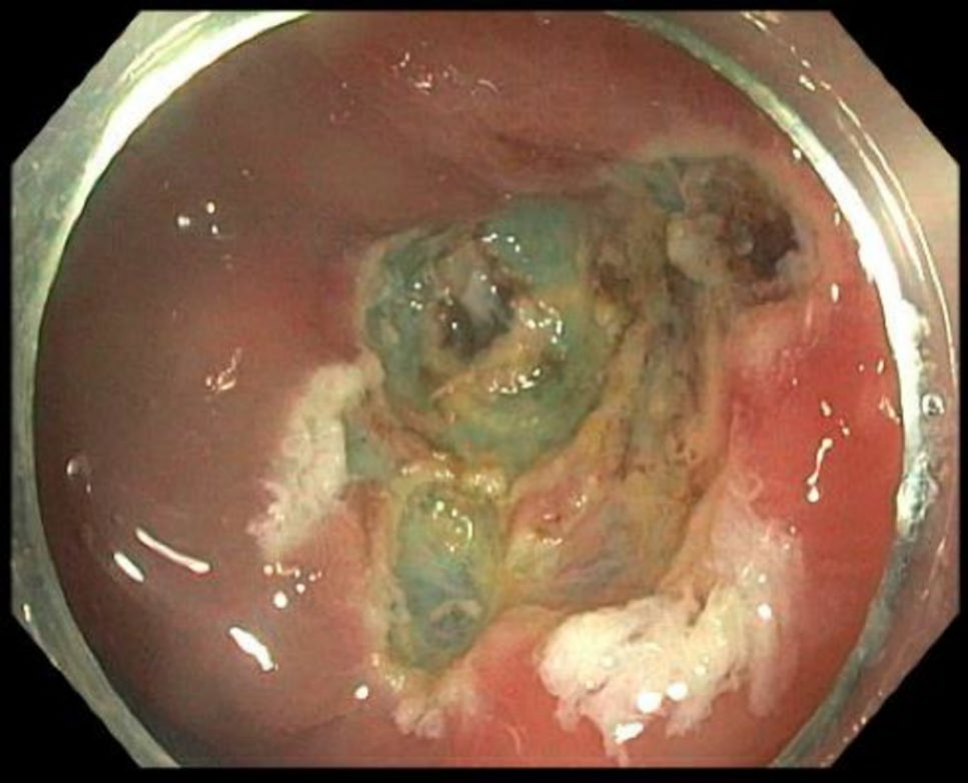
Appearance after ESD resection of an appendiceal orifice mucosal lesion

For an ESD approach, a slim size colonoscope (for example, the EVIS EXERA III PCFH190L/ I colonovideoscope, Olympus®, Massachusetts, USA) is used to identify the lesion at the appendiceal orifice. A soft transparent hood (for example, Distal Attachment, Olympus®, Massachusetts, USA) is attached to the tip of the colonoscope. This allows for traction and retraction, helping to stretch a fold or lifting a lesion with the edge. An injectable agent (for example, ORISE Gel Submucosal Lifting Agent, Boston Scientific®, Massachusetts, USA) is used to lift the mucosa and submucosa away from the muscularis propria. Specifically, for AO lesions, we begin by injecting centrally within the AO. This allows the lesion to pop out of the appendix, rather than being pushed in if we were to start the injection on the periphery of the lesion furthest away from the AO. This everting technique is the key step in successfully managing AO lesions using advanced endoluminal approaches. The volume of injectate can vary widely by lesion size, tissue resistance and degree of fibrosis. Next, a circumferential incision is made around the lesion into the submucosa using a dual knife, any other endo-knife or snare tip cutting and ensuring that a margin of normal mucosa is included in the resection. The dissection is then continued staying in the submucosal plane and avoiding injury to the muscularis mucosa. Lifting injections can be repeated during the course of the dissection as needed. Once the lesion is completely resected, the resection field is closely inspected for any defects and/or bleeding, both of which can be addressed with endoscopic hemoclips. A potential pitfall during clipping is occluding the appendiceal lumen; thus, we ensure that the appendiceal lumen is not occluded by the clip to prevent potential appendiceal inflammation after the procedure. Mild bleeding can be addressed with heat via the dual knife or coagulation forceps, but it is important to prevent creating a defect into the muscular layer. Figures 1, 2, and 3 show an example of an AO lesion under normal light (Fig. 1) and narrow band imaging (NBI) (Fig. 2) before ESD and post-ESD (Fig. 3).

A similar approach was used for cases completed by EMR or hybrid ESD (combining both ESD and EMR) [10]. Hybrid ESD involves submucosal injection followed by use of a knife probe to create a working edge, as in ESD, to which then a snare can be applied as in EMR. We have previously described our approach to CELS [11].

## Results

A total of 41 patients underwent AOMN resection. These 41 lesions represented 4% of the 1005 colorectal lesions resected with advanced endoscopic techniques during the study time period. Patient and lesion factors are described in Table 1. The median age of the study patients was 65 years (IQR 60–73) and 54% were male. Most patients carried significant comorbidities with the majority (87%) of patients being categorized as ASA (American Society of Anesthesiologist classification) score 2 or greater. The median lesion size was 20 mm (IQR 14.5–27.5). The majority of the lesions were Paris type 0-IIa (42%) or Is (22%). There were 3 lesions that had undergone previous submucosal injections at time of previous endoscopy (data not shown in tabular form).

**Table 1:** Patient and lesion factors

Age, years	65 (60–73)
Sex	
Male	22 (53.7%)
Female	19 (46.3%)
BMI, kg/m^2^	28.0 (24.4–30.4)
ASA	
1	5 (12.2%)
2	28 (68.3%)
3	8 (19.5%)
Lesion size (mm)	20.0 (14.5–27.5)
Paris type	
0-IIa flat elevation	17 (41.5%)
0-IIa + c flat elevation + central depression	3 (7.3%)
Is sessile	9 (22.0%)
Other/not recorded	12 (29.2%)
Kudo	
II	5 (12.2%)
IIIs	13 (31.7%)
IIIL	7 (17.1%)
Other/not recorded	16 (39.0%)

Reported as frequency (%) or median (interquartile range)

Procedure factors and outcomes are described in Table 2. Most lesions were resected with hybrid ESD (39%) or ESD (34%) with the remaining being approached by straight EMR (15%) or CELS (12%). The dissection was completed en bloc in 21 (51%) patients; all lesions were removed completely clinically. The overall median procedure time was 63 (IQR 29.8–97.3) minutes with the actual dissection time being a median of 18 (IQR 12–52.5) minutes. Cases were performed in the endoscopy suite 59% of the time; the remaining cases were completed in the operating room (including all CELS cases). Endoscopic hemoclips were utilized in 34% of lesions. No patients had an intra-procedural perforation.

**Table 2:** Procedure factors and outcomes

Procedure type	
ESD	14 (34.1%)
EMR	6 (14.6%)
Hybrid ESD	16 (39.1%)
ESD + CELS	5 (12.2%)
Overall procedure time (minutes)	63.0 (29.8–97.3)
Dissection time (minutes)	18.0 (12.0–52.5)
Procedure location	
Endoscopy suite	24 (58.5%)
Operating room	17 (41.5%)
Resection specimen	
Piecemeal	20 (48.8%)
En bloc	21 (51.2%)
Endoscopic hemoclip utilization	14 (34%)
Final pathology	
Sessile serrated adenoma	20 (48.8%)
Tubular adenoma	10 (24.4%)
Tubulovillous adenoma	6 (14.6%)
Inflammatory changes	1 (2.4%)
Hyperplastic polyp	1 (2.4%)
Other	3 (7.4%)
Discharge	
Same day	31 (75.6%)
Post-procedure day #1	9 (22.0%)
30-day complications	
No	40 (97.6%)
Yes	1 (2.4%)

Reported as frequency (%) or median (interquartile range)

Pathology revealed 20 (49%) sessile-serrated lesions, 10 (24%) tubular adenomas, and 6 (15%) tubulovillous adenomas; the remaining polyps included hyperplastic polyps and inflammatory changes. One tubulovillous adenoma had high-grade dysplasia. One lesion (under the other category) was endoscopically described as a submucosal nodule. After advanced endoscopic resection, pathology showed mucin but no dysplastic tissue; note that prior to resection there was no specific concern to suggest a mucinous neoplasm which would have led to a different treatment algorithm altogether. Following cross-sectional imaging and tumor board review, the patient underwent an appendectomy with cecal wedge resection and pathology revealed a low-grade appendiceal mucinous neoplasm that did not require any further treatment.

Post-procedure, one patient was admitted with post-polypectomy abdominal pain. The patient’s CT of the abdomen and pelvis was concerning for a possible micro-perforation and the patient was observed for 2 days with no intervention. The other 40 patients (98%) did not experience any post-procedure complications within 30 days. One (2%) patient with a sessile-serrated adenoma experienced a recurrence after EMR which was re-resected with EMR. Patients were followed up for a median of 8 (0–48) months.

## Discussion

The current study reports our approach and experience with advanced endoscopic techniques for AOMN which were referred after being deemed not amenable to conventional endoscopic techniques. All 41 lesions were clinically completely resected and nearly all had benign final pathology results. Only one lesion (2%) had concerning features in pathology, in this case mucin, prompting further resection after multi-disciplinary discussion and ultimately revealing a low-grade appendiceal mucinous neoplasm. Likewise, only one (2%) patient experienced a recurrence which was subsequently managed by EMR. As such, this organ preservation approach appears safe from an oncologic standpoint. From a complication standpoint, this approach is also safe with only one patient (2%) experiencing a self-limited post-operative complication, a micro-perforation, which was managed non-operatively and with a short hospital stay.

Our results support a growing body of literature demonstrating that most polyps that are unresectable by conventional endoscopic techniques, but are otherwise benign appearing, are in most cases indeed benign. For instance, in a cohort of 439 patients referred for colectomy secondary to endoscopic diagnosis of benign adenoma not amenable to conventional endoscopic removal, only 8% showed evidence of cancer in final pathology [12]. Endoscopic features concerning for malignancy include ulcerations, irregular pit pattern, firm or fixed mobility on manipulation, or friability with easy bleeding [12]. One patient in our cohort had polyp recurrence which was managed with EMR. A Korean multicenter study of AO lesions resected with various endoscopic techniques including ESD and EMR showed a recurrence rate of 15%; however, the majority were able to be managed with further endoscopic techniques [13]. Furthermore, there is potential for preventing substantial morbidity and mortality by utilizing advanced endoscopic techniques rather than surgical resection. It has been previously demonstrated that colectomy and proctectomy for non-malignant colorectal polyps is associated with approximately 1% in-hospital mortality and up to 25% of patients suffer perioperative morbidity [3]. Factors associated with increased mortality included older age and burden of comorbidities in the aforementioned study. Our cohort of patients is indeed older and comorbid; a group which is prone to complications from abdominal surgeries. A study using NSQIP (National Surgical Quality Improvement Program) data similarly showed 1% mortality among patients who underwent elective surgical resection of non-malignant polyps and lower, yet still substantial, morbidity with 14% experiencing major post-operative events [14]. While in real-world practice, we do know that some surgeons may tackle these AO lesions with a partial or wedge cecal resection with en bloc appendectomy, rather than cacectomy or right colectomy with its known risks. Such surgeries may be completed in a shorter time when compared to the overall procedure time in the current study. Also, partial or wedge cecal resection with appendectomy can be considered a safer procedure when compared to right hemicolectomy, whenever there is a limitation to access for advanced endoscopy. It is our practice first we start endoscopically and complete either EMR or ESD for carpeted polyps expanding outside the orifice of the appendix into the cecum and resect the adenoma tissue down to the submucosal/muscle layer. If the adenoma is expanding deep into the appendiceal lumen and is in continuity with the appendix, endoscopically these lesions may not be completely removed. Thus, an appendectomy with the base of the cecum may be required. In such instances, a combined endoscopic laparoscopic approach would enable complete R0 resection and leave no adenoma tissue behind at the cecal margin is assured by colonoscopic visualization of the cecal base.

While ESD and EMR are also associated with periprocedural complication rates, these are much lower than for resections. A meta-analysis of endoscopic resection of large colorectal polyps showed that perforation occurred in 1.5% of polyps and bleeding occurred in 6.5% although periprocedural and post-procedural bleeding were not always defined [15]. In a more recent prospective multicenter North American study, perforation was noted in 4.7% of colon ESD procedures and delayed bleeding occurred in 1.9% [7]. In our cohort, endoscopic hemoclips were utilized in 34% of patients. These are used for areas with concern for bleeding (active or potential for delayed bleeding) or slightly deeper tissue defects which could be prone to micro-perforation or delayed bleeding as well. Only one patient in our cohort experienced a micro-perforation which was managed non-operatively and no patient experiences delayed bleeding. While we cannot provide any concrete evidence as to why our complication rates are quite low, we suspect that provider level of experience likely accounts for this. Moreover, ESD has been shown to be more cost-effective than segmental resections [16].

AOMN pose various challenges to the skilled advanced endoscopist/surgeon: geometrically difficult area, thin-walled cecum, and excision of a lesion within an orifice which may partially involve the circumference or could even be ‘donut-like’ [9, 17, 18]. In a Japanese study of 76 AO lesions approached with ESD, the rates of perforation and post-operative bleeding were both 1.3% [17]. In this study, 2 patients (3%) developed post-ESD acute appendicitis and the authors suggest that clip closure ultimately narrowing or closing the AO/lumen could be a risk. Note that several case studies have also reported appendicitis following conventional and advanced endoscopic techniques in the cecum [19, 20]. In our patient cohort, there were no associated acute appendicitis cases, although ensuring that the AO is not closed off by the procedure is a key part of our technique.

The present study has several limitations including our small patient cohort. This is a retrospective analysis of prospectively collected data; we cannot make any direct comparisons to alternative management strategies. Additionally, there is certainly bias with regards to what lesions are referred to our program. Furthermore, the lesions which have specific follow-up at our institution are not random and as such our surveillance data is limited. Given our referral patterns, most patients are referred by an outside endoscopist to whom the patient is referred back for follow-up. The patients that have ongoing follow-up at our institution are either originally a patient here, had a specific concern during endoscopy prompting follow-up specifically here, or had a recurrence and were once again referred back. Lastly, these results are not generalizable to all endoscopists/surgeons; these are the outcomes of a skilled and experienced advanced surgical endoscopist. Nevertheless, we demonstrated that AOMN can be successfully managed with advanced endoscopic techniques safely from an oncologic and complication standpoint. Changes in treatment paradigms and enriched education/training efforts will be necessary in order to improve patient access to advanced endoscopic techniques and organ preservation.

## Conclusion

Advanced endoscopic interventions for lesions involving the AO can be performed with a low rate of complications and recurrence. While conventionally premalignant lesions at the AO are often treated with surgical resections, whether a cecal wedge with en bloc appendectomy or an actual ileocolic or right hemicolectomy, advanced endoscopic interventions are an alternative approach with promising results allowing for organ preservation.
